# A Prospective Cohort Study to Evaluate Needle Passes Using a Portable Ultrasound Device versus Traditional Landmark Approach for Epidural Anesthesia in a Busy Obstetric Tertiary Care Center 

**DOI:** 10.24908/pocus.v8i2.16298

**Published:** 2023-11-27

**Authors:** Antonio Gonzalez Fiol, Pedro Acevedo Rodriguez, Xiwen Zhao, Robert Gaiser, Adriana Herrera, Aymen Alian

**Affiliations:** 1 Department of Anesthesiology, Yale School of Medicine New Haven, Connecticut

**Keywords:** Neuraxial anesthesia, Regional anesthesia, Pregnancy, ultrasound guided neuraxial anesthesia, handheld ultrasound, portable ultrasound

## Abstract

Despite its many cited benefits, ultrasound guidance for neuraxial procedures is not widespread in anesthesiology. Some cited limitations include device cost and accessibility. We test the hypothesis that a handheld and relatively inexpensive ultrasound can improve neuraxial proficiency (e.g., decreased needle manipulations and block time). This prospective study compared the number of needle passes, redirections, and procedural time between epidural placed with a handheld ultrasound versus landmarks. Needle passes and attempts were defined as the number of times the Tuhoy needle was redirected, and the times skin was punctured (re-insertion). Procedural time was defined as the time from local anesthetic infiltration until loss of resistance was obtained. The impact of level of training and accuracy of the device were also analyzed. 302 patients receiving labor epidural were included in the study. No difference in body mass index (BMI) nor distribution of level of training was noted between the groups. Regression analysis adjusted for BMI demonstrated a decrease in needle passes (-1.75 (95% CI -2.62, -0.89), p < 0.001), needle attempts (-0.51 (95% CI -0.97, -0.04), p = 0.032) and procedural time (-154.67s 95% CI -303.49s, -5.85s), p = 0.042) when a handheld ultrasound was utilized. The mean (95% Confidence interval) difference between needle depth and ultrasound depth was 0.39 cm (0.32, 0.46), p < 0.001. The use of a handheld device resulted in statistically significant decrease of needle manipulations and block time. More research is needed to evaluate the impact of and increase in accessibility of ultrasound technology.

## Introduction

Neuraxial anesthesia procedures are one of the few procedures anesthesiologists perform that mainly rely on proceduralists tactile feedback without needle visualization. Many of the techniques that once were performed by utilizing anatomic landmark (e.g., central line placement) or nerve block using stimulators are now performed with ultrasound (US) guidance. The first description of US guidance for neuraxial anesthesia occurred in 1980, demonstrating good correlation between estimated depth from skin to epidural space 1, 2. Proponents of ultrasound-guided neuraxial block, including the National Institute for Health and Care Excellence (NICE) have cited improved efficacy of block placement (e.g., first attempt success), decreased epidural catheter failure rate, and better localization of the epidural space in patients with poor anatomical landmarks or abnormal anatomy (e.g., scoliosis) 3, 4, 5, 6.

Despite the benefits of US guided neuraxial blockade, its use has not gained traction over the years. Some limitations to the incorporation of its use includes time constraints, lack of formal training, limited availability, high cost of ultrasound devices, ease of use and storage space 7, 8. Technological advancements, such as the utilization of capacitive micro-machined ultrasound transducers (CMUTs) instead of piezoelectric crystals has allowed for the creation of more portable and affordable devices. The Butterfly iQ ultrasound (Butterfly Network, Burlington, MA) device is a handheld that utilizes this technology and the image quality produced by their device is comparable to that of cart-based US devices, particularly for basic obstetric and anesthesiology ultrasound imaging 9. The primary aim of this study was to evaluate the number of needle passes (redirections). Secondary outcomes include feasibility, time from start of procedure to loss of resistance, and number of needle attempts. Secondary outcomes included accuracy of the device (defined as the difference between the actual depth from skin to epidural space minus the estimated depth from skin to epidural space calculated by the Butterfly iQ device) and impact of level of training over the primary and secondary outcomes with and without neuraxial US guidance. 

## Methods

After approval by the Internal Review Board (IRB) at Yale New Haven Hospital (ID# - 2000030405), this prospective cohort study was conducted from September 2021 to May 2022. The strengthening the reporting of observational studies in epidemiology (STROBE) guidelines were followed. Laboring women requesting epidural analgesia were recruited after giving verbal consent. Patients were assigned to one of two groups: landmark, or US guided neuraxial placement according to attending anesthesiologist preference. Inclusion criteria was laboring parturient ASA Physical Status I-III. Exclusion criteria were patients with a contraindication for neuraxial anesthesia (e.g., coagulopathy), known diagnosis of scoliosis, or history of spinal surgery. 

### Ultrasound and landmark assessment 

**Figure 1:**
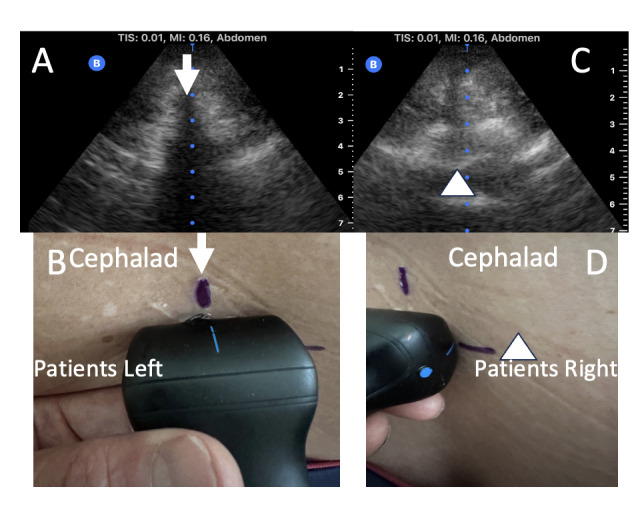
Image A demonstrates the acoustic shadow that characterizes the spinous process (arrow). Image B depicts the marking of the patients midline (arrow). Image C. can be obtained by moving the ultrasound in a cephalad or caudad position after obtaining image A. The arrowhead tip is pointing at the posterior complex which is composed of the ligamentum flavum and the dura. This view is utilized to mark the interspace as demonstrated by Image D, depicting the marking of the interspace (arrowhead).

**Figure 2:**
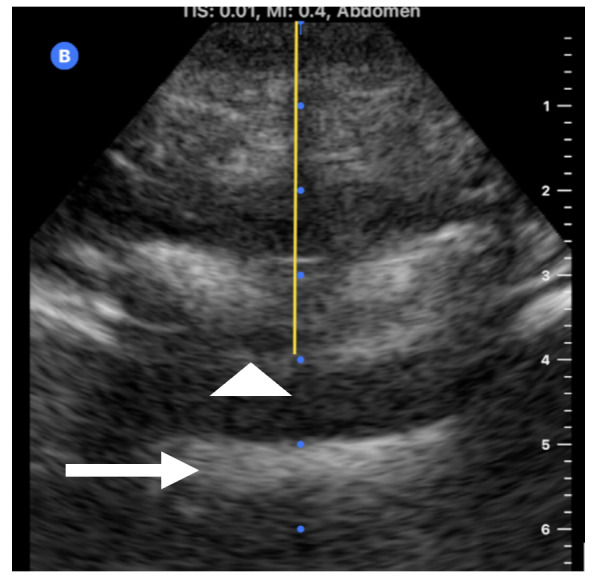
Butterfly US image showing posterior complex (arrow head) that represents Ligamentum flavum and dura, and anterior complex (arrow) which represents vertebral body. Yellow line represents the caliper measuring the distance between skin and posterior complex.

Patients were placed in the seated position and instructed to flex their lumbar spine. For patients in the landmark approach (LM) cohort, the iliac crest was palpated, and spaces were determined at that intersection point. An anesthesia provider (resident, fellow or Certified Registered Nurse Anesthetists) level of training (LOT) 1 – 4 performed the labor epidural utilizing a loss of resistance technique at the level that was the most favorable to them. Level of training was defined according to years of experience in the field of anesthesia, with residents in the first clinical year of anesthesia being LOT 1, LOT 2 or 3 if on their second or third year, respectively. Fellows, attendings and Certified Registered Nurse Anesthetists (CRNAs) were considered – LOT 4, given at least 4 years of experience in the field. In the ultrasound cohort, the iliac crest was identified, and a transverse ultrasound scan was performed starting at the intercristal line (also known as the Tuffier’s line). The US technique was performed utilizing a handheld second-generation Butterfly iQ + US device (Figure 1), with the abdomen preset, and performed by one of two researchers (AG-F or AA), both with > 5 years of experience performing US guided neuraxial anesthesia procedures. The US probe was moved vertically upward until the posterior and anterior complexes were clearly visualized. A line was drawn at the midpoint of the probe in the horizontal and vertical plane as previously described by Balki et al 10. The image was frozen, and the built-in caliper was utilized to measure (to the 2nd tenth of a decimal point) the estimated distance from skin to the posterior complex (Figure 2). To limit collection bias, the times and number of needle passes and attempts (for both groups) were recorded by a third person not involved in the care of the patient.

### Epidural procedure

With the patient in the sitting position and with their lumbar spine flexed, the back was cleaned and draped. After infiltration of the skin with a 1% solution lidocaine, a 17-gauge Tuohy needle (8.9 cm) with 1 cm markings was utilized using a loss of resistance to saline or air technique. In the landmark group anesthesia providers palpated the patients back and proceeded at the location they thought were most easily palpated. In the ultrasound group, the anesthesia providers, who were blinded to the estimated distance, were asked to refrain from palpating and to proceed with the epidural at the intersection point between the horizontal and vertical markings. Upon loss of resistance the depth from skin to epidural space was visually estimated to the tenth of a decimal point. The primary outcome of needle passes was defined as any change in the angle of the Tuohy needle. Secondary outcomes for time for epidural placement (procedure time) were defined as time from local anesthetic until time at which loss of resistance was achieved, and a needle attempt was defined as the number of times the needle was re-inserted at the skin. 

### Statistical analysis 

Based on previous studies that demonstrated a mean number of 4 needle passes (standard deviation of 2) in patients with “difficult backs”, and considering a difference of two passes between groups as clinically significant, a calculated 20 patients per group was needed to provide a power of 80% at a two-sided significance level of 0.05 11, 12. Given that one of these studies demonstrated to be underpowered, we were unaware of our mean (SD) for number of needle of passes, and to account for the differences in level of training we opted to increase the number of patients to 35 per each level of training patients per group, and up to 310 patients total would be recruited to account for missing data or protocol violations. A total of 302 patients were included in the data analysis (150 US vs. 152 LM). Demographics characteristics were summarized by mean (SD) for continuous variables and count (%) for categorical variables. The difference between US and LM were tested using 2 sample test, Wilcoxon rank sum test, person’s chi square test, or fisher’s exact test when appropriate. We also run linear regression models to study association of US to attempts, passes, and block time after adjusting for the BMI and level of training. All tests were 2-sided with a significance level of 0.05. All statistical analysis were conducted in R version 4.0.2 (R core Team). 

## Results

We enrolled 302 patients during the study period. One hundred fifty patients were included in the US group and 152 in the LM group. No statistically significant difference was noted regarding body mass index (BMI) between the groups. Table 1 summarizes the patients and level of training characteristics data as well as the differences in the number of needle pass, attempts and time to perform the block and percentage of success upon first attempt and pass. The unadjusted number of needle pass attempts and time to perform the block and percentage of success upon first attempt and pass by level of training are summarized in Table 2. 

**Table 1:** Patient body mass index, level of training distribution and overall impact of handheld ultrasound over needle attempts, passes and block time.

**Characteristic**	**Landmark group (LM)**	**Ultrasound group (US)**	p-value^*^
BMI (Kg/m^2^)			
Mean (SD)	32.86 (7.78)	32.82 (7.99)	> 0.9
Level of training	n/N (%)	-	0.6
1	3/152 (2%)	3/152 (2%)	-
2	41/152 (27%)	35/150 (23%)	-
3	58/152 (38%)	51/150 (34%)	-
4	50/152 (33%)	61/150 (41%)	-
First needle pass success	70/152 (46%)	113/150 (75%)	<0.001
First needle attempt success	91/152 (60%)	126/150 (84%)	<0.001
Needle Passes	-	-	<0.001
Mean (SD)	2.80 (2.45)	1.43 (0.55)	-
Median (IQR)	2.00 (1.00 – 4.00)	1.00 (1.00 – 1.00)	-
Range	2.00 (1.00 – 13.00)	1.00 (1.00 – 6.00)	-
Needle Attempts	-	-	<0.001
Mean (SD)	1.79 (1.30)	1.22 (0.55)	-
Median (IQR)	1.00 (1.00 – 2.00)	1.00 (1.00 – 1.00)	-
Range	1 - 7	1 - 4	-
Block time in seconds	-	-	<0.001
Mean (SD)	342.20 (414.62)	184 (174.28)	-
Median (IQR)	215 (98.75 – 447.00)	120 (84.25 – 193.50)	-
BMI—Body mass index; IQR – Interquartile ratio; LM – landmark group; US – handheld ultrasound group; SD – Standard deviation; ^*^Welch Two Sample t-test; Fisher's exact test; Pearson's Chi-squared test.			

**Table 2:** Impact of level of training (LOT) over needle passes, attempts and block time.

** -**	**LM group**	**US group**	**p-value**
Needle Passes			
LOT 2	3.39 (2.54)	1.63 (1.09)	<0.001
LOT 3	2.90 (2.70)	1.31 (0.97)	<0.001
LOT 4	1.98 (1.71)	1.41 (0.78)	0.033
Needle attempts			
LOT 2	1.88 (1.31)	1.31 (0.63)	0.017
LOT 3	1.74 (1.29)	1.14 (0.45)	0.001
LOT 4	1.62 (1.09)	1.25 (0.60)	0.032
Procedure time in seconds			
LOT 2	402.78 (291.10)	232.82 (192.93)	0.003
LOT 3	328.66 (494.62)	132.41 (106.15)	0.005
LOT 4	264.74 (357.63)	203.28 (201.88)	0.3
US – Handheld Ultrasound group. LM – landmark group; LOT2 – resident with 2 clinical years of experience; LOT3 – resident with 3 years of clinical experience; LOT4 – anesthesiology provider with at least 4 years of experience (e.g., Attending, fellow) or Certified Registered Nurse .			

For the primary outcome of needle pass, after adjusting for BMI, the regression analysis demonstrated that the use of US resulted in a decrease in needle passes of -1.75 (95% CI -2.62, -0.89), p < 0.001. For the secondary outcomes of needle attempts and procedure time, the use of US also resulted in a reduction in these variables ([-0.51 (95% CI -0.97, -0.04), p = 0.032] and [-154.67 s 95% CI -303.49s, -5.85s), p = 0.042], respectively). When examining level of training, the regression analysis demonstrated that only LOT4 with and without the aid of US resulted in a decrease in number of needle pass [1.17 (95% CI 0.06 – 2.28), p = 0.039 and -1.39 (95% CI -2.16, -0.61), p < 0.001], respectively (Table 3). 

**Table 3:** Summary of the statistically significant predictors noted on the regression analysis.

Predictors	Estimates (Confidence interval)	p-value
Needle passes		
Intercept*	3.18 (2.56 – 3.80)	<0.001
BMI	0.03 (0.00 – 0.06)	0.033
LOT4	-1.39 (-2.16 - **-**0.061)	<0.001
US	-1.75 (-2.62- **-**0.89)	<0.001
US and LOT4	1.17 (0.06 – 2.28)	0.039
Needle attempts		
Intercept*	1.80 (1.47 – 2.14)	<0.001
US	-0.51 (-0.97 - **-**0.04)	0.032
Block time in seconds		
Intercept*	361.17 (254.39 – 467.95)	<0.001
BMI	4.89 (0.20 – 9.58)	0.041
US	-154.67 (-303.49 - -5.85)	0.042
* Level of training 2 – residents with at least 2 years of clinical experience were defined as the intercept. BMI – body mass index; LOT4 – anesthesiology provider with at least 4 years of experience (e.g., Attending, fellow) or Certified Registered Nurse.		

When further evaluating procedure time, the mean (SD) time for US image acquisition was 82.10 (65.25) s. After factoring in the US-total block time (scanning + block time), the US device group time from local anesthetic to loss of resistance was faster than that of the LM group (266.06 (181.33) and 342.20 (414.62) seconds, p = 0.04, respectively). The regression analysis model (adjusted for BMI) demonstrated that BMI added 0.03 (95% CI 0.00, 0.06), p = 0.033 passes and approximately 5 seconds (95% CI 0.20, 9.58), p = 0.041, for every 1 kg/m2 above 25 kg/m2. Furthermore, when all variables (e.g., BMI, LOT, use of US, LOT + use of US) were compared to LOT 2, the only factor that consistently provided statistically significant difference in the number of passes, attempts and block time was the use of US. Overall, the variables herein reported (BMI, level of training, use of ultrasound) explain less than 20% of the variance when evaluating their impact over needle passes (R2/ R2 adjusted 0.165/0.147), attempts (R2/ R2 adjusted 0.072/0.052) and block time (R2/ R2 adjusted 0.084/0.064). Lastly, the mean difference between needle depth and US device estimated depth was 0.39 cm (95% CI: 0.32, 0.46), p < 0.001.

## Discussion

The most important finding in our study was that the use of ultrasound halved the number of needle passes and decreased the overall number of attempts and procedural time for epidural anesthesia. Our first needle pass and attempt success rate increased by 84% and 40%, respectively. These results echo those previously reported in several studies^12^, ^13^, ^14^, ^15^, ^16^ and meta-analysis 17, 18, 19. Time constraint is usually mentioned as a limitation for the use of US 3. In our study the mean scanning time was < 2min, and the addition of this time is unlikely to be of important clinical significance to the total block time. Although not evaluated in our study, other studies suggest that this decrease in needle passes and attempts has been associated with a 50% reduction technical failure (relative risk 0.51; 95% CI 0.32 – 0.80), improved maternal satisfaction, better analgesia and a decrease in reported headaches, and back pain 17, 18, 19. These benefits may not be as evident in the concomitant presence of palpable anatomy and an experienced sonographer and provider 17, 18, 19.

The use of US guidance is more likely to yield significant improvement in the presence of abnormal or poorly palpable anatomy 5, 6. Yet, it is important to recognize that several experts in the field of neuraxial US guidance agree that this is a technique that requires practice, and perhaps unlikely to yield noticeable outcomes without a priori normal anatomy pattern recognition and marking skills 3, 20. A study by Arzola et al. 21, demonstrated that when US imaging acquisition and marking was performed by relatively inexperienced providers, there was no benefits to the use of this technology. Our study controlled for the experience of the sonographer, and it confirms an overall improvement in block insertion efficiency. As noted in Table 2, all LOT benefited from the use of US, yet this observation does not account for the weight experience may carry. The regression analysis (Table 3) suggests that only the most experienced providers (LOT4) were able to decrease their number of needle passes with and without the use of US. These results suggests that LOT4 providers can make accurate anatomical determinations of midline and needle adjustments with and without US guidance, a finding that agrees with previous studies that suggest minimal benefit for the use of US when experienced providers perform the block 11.

Additionally, we found that the depth from skin to epidural depth was able to be estimated using handheld US within 0.46 cm. The mean and confidence intervals for the difference between needle depth and estimated US depth described in our study are in agreement with those previously cited in several studies 7, 10, 19, 22. Our findings also confirm that the estimated depth by US tends to underestimates the actual needle depth 3, 7, 21, 23. This discrepancy can be explained by a divergence between the US beam and the needle trajectory, soft tissue compression with the US probe, skin deformation by the needle, and inadvertent deviation of the needle from midline 4, 10. Overall, this technical limitation may provide an additional margin of safety for the provider 7.

Our study presents several limitations, the prospective and non-randomized study design may have predisposed to selection bias. The recording of the times and number of needle passes and attempts were recorded by a third person not involved in the care of the patient to limit data collection bias. Performer bias cannot be ruled out, it is possible that residents performed faster blocks in the US group thinking that this was the expected result, on the other hand LOT4 group may have skeptically approached the US markings. This may explain that overall LOT3 were faster than LOT4 when US was utilized. It is also possible that the most experienced providers (LOT 4), may have been requested to do blocks more for patients with suspected poorly palpable anatomy (e.g., history of a previous difficult block). Other limitations include classification of patients according to their anatomy (e.g., palpable versus poorly palpable). Another important limitation is that the needle depth was estimated, not measured. Lastly, we did not document early or late failed labor epidural which could have added information regarding the benefits or lack thereof for the use of US in establishing neuraxial analgesia.

In conclusion, the use of US in our study resulted in a decrease in the procedural time, and number of needle passes and attempts needed to locate the epidural space. These benefits were less evident when the blocks were performed by the most experienced provider, particularly in terms of procedural time. Our study confirms that the use of handheld US, is a portable alternative to cart-based devices, and can be a useful tool in neuraxial anesthesia placement in a busy tertiary care center. Although our results regarding accuracy of depth are limited, our results suggest that the handheld US can provide information regarding depth within the previously cited margin of accuracy for cart-based and other handheld devices. Further studies are needed to assess handheld US guided epidural anesthesia’s impact on failed labor epidural rate and patient satisfaction. 

## Disclosures

The authors report no relevant disclosures related to this work.
